# Restoration of intrinsic hand function by superficial radial nerve: an anatomical study

**DOI:** 10.1186/s12891-023-06758-3

**Published:** 2023-08-02

**Authors:** Shuo Liang, Yu-Zhou Liu, Xiao-Qian Hu, Xin Zhao, Jie Lao

**Affiliations:** 1 Department of Hand Surgery, Huashan Hospital, Fudan University, Shanghai, China; 2 Key Laboratory of Hand Reconstruction, Ministry of Health, Shanghai, China; 3 Shanghai Key Laboratory of Peripheral Nerve and Microsurgery, Shanghai, China

**Keywords:** Nerve regeneration, Brachial plexus avulsion injury, Contralateral C7 transfer, Motor branch of ulnar nerve, Superficial radial nerve, Intrinsic hand function, Anatomical study, Modified surgery

## Abstract

**Background:**

The contralateral seventh cervical (cC7) nerve root transfer represents a cornerstone technique in treating total brachial plexus avulsion injury. Traditional cC7 procedures employ the entire ulnar nerve as a graft, which inevitably compromises its restorative capacity.

**Objective:**

Our cadaveric study seeks to assess this innovative approach aimed at preserving the motor branch of the ulnar nerve (MBUN). This new method aims to enable future repair stages, using the superficial radial nerve (SRN) as a bridge connecting cC7 and MBUN.

**Methods:**

We undertook a comprehensive dissection of ten adult cadavers, generously provided by the Department of Anatomy, Histology, and Embryology at Fudan University, China. It allowed us to evaluate the feasibility of our proposed technique. For this study, we harvested only the dorsal and superficial branches of the ulnar nerve, as well as the SRN, to establish connections between the cC7 nerve and recipient nerves (both the median nerve and MBUN). We meticulously dissected the SRN and the motor and sensory branches of the ulnar nerve. Measurements were made from the reverse point of the SRN to the wrist flexion crease and the coaptation point of the SRN and MBUN. Additionally, we traced the MBUN from distal to proximal ends, recording its maximum length. We also measured the diameters of the nerve branches and tallied the number of axons.

**Results:**

Our modified approach proved technically viable in all examined limbs. The distances from the reverse point of the SRN to the wrist flexion crease were 8.24 ± 1.80 cm and to the coaptation point were 6.60 ± 1.75 cm. The maximum length of the MBUN was 7.62 ± 1.03 cm. The average axon diameters in the MBUN and the anterior and posterior branches of the SRN were 1.88 ± 0.42 mm、1.56 ± 0.38 mm、2.02 ± 0.41 mm,respectively. The corresponding mean numbers of axons were 1426.60 ± 331.39 and 721.50 ± 138.22, and 741.90 ± 171.34, respectively.

**Conclusion:**

The SRN demonstrated the potential to be transferred to the MBUN without necessitating a nerve graft. A potential advantage of this modification is preserving the MBUN’s recovery potential.

## Introduction

Brachial plexus injury, particularly total brachial plexus avulsion injury, is a major cause of paralysis and sensory loss in the affected limb. Surgical nerve transfer remains the primary approach for the restoration of motor and sensory function [4–6, 9]. However, donor nerves for total brachial plexus injuries are scarce. In 1991, Gu et al. pioneered the innovative surgical procedure of cC7 transfer for treating total brachial plexus avulsion injury [10], which has since been widely adopted by many centers [8, 19, 20, 22]. The cC7 nerve, with an average total axonal count of approximately 27,000, offers a plentiful source of nerve fiber from the unaffected side without causing permanent morbidity in the healthy limb [7]. The two-stage surgical process first transfers the cC7 to the ulnar nerve, which is then anastomosed with the median nerve or another recipient nerve in the second stage. The cC7 can reconstruct finger sensation and motor function of the shoulder, elbow, and hand [4].

Research has shown that the intrinsic hand muscles play a crucial role in finger flexion and fine hand movements [2]. Therefore, the heart of upper limb function lies in the performance of the intrinsic hand muscles: thumb opposition, flexion of metacarpophalangeal joints, and extension of interphalangeal joints [1, 2, 17]. The adductor pollicis, hypothenar, interosseus, and the third and fourth lumbrical muscles are innervated by the MBUN. Consequently, the restoration of fine hand function hinges on MBUN repair. Yet, in traditional procedures, the entire segment of the ulnar nerve is harvested as a graft to bridge the gap between the median nerve and cC7, which eliminates the possibility of MBUN recovery.

For combined high median and ulnar nerve injuries, using the anterior interosseous nerve as a graft to connect the donor nerve to MBUN has been reported [16]. This experience suggests that MBUN repair is possible. Furthermore, Rui et al. conducted an anatomical study suggesting that the pronator quadratus motor branch could be transferred to the MBUN without a nerve graft, subsequent to the cC7 [18]. There are also reports of the cC7 using the medial antebrachial cutaneous nerve as a bridge to MBUN [11]. These studies inspire the idea that if the MBUN is severed near the wrist, allowing the MBUN to become the target nerve of the contralateral C7 nerve, there may be potential for the regeneration of the intrinsic muscles. Simultaneously, it does not impede the primary trunk of the ulnar nerve acting as the bridge nerve for the contralateral C7 transfer.

**Fig. 1:**
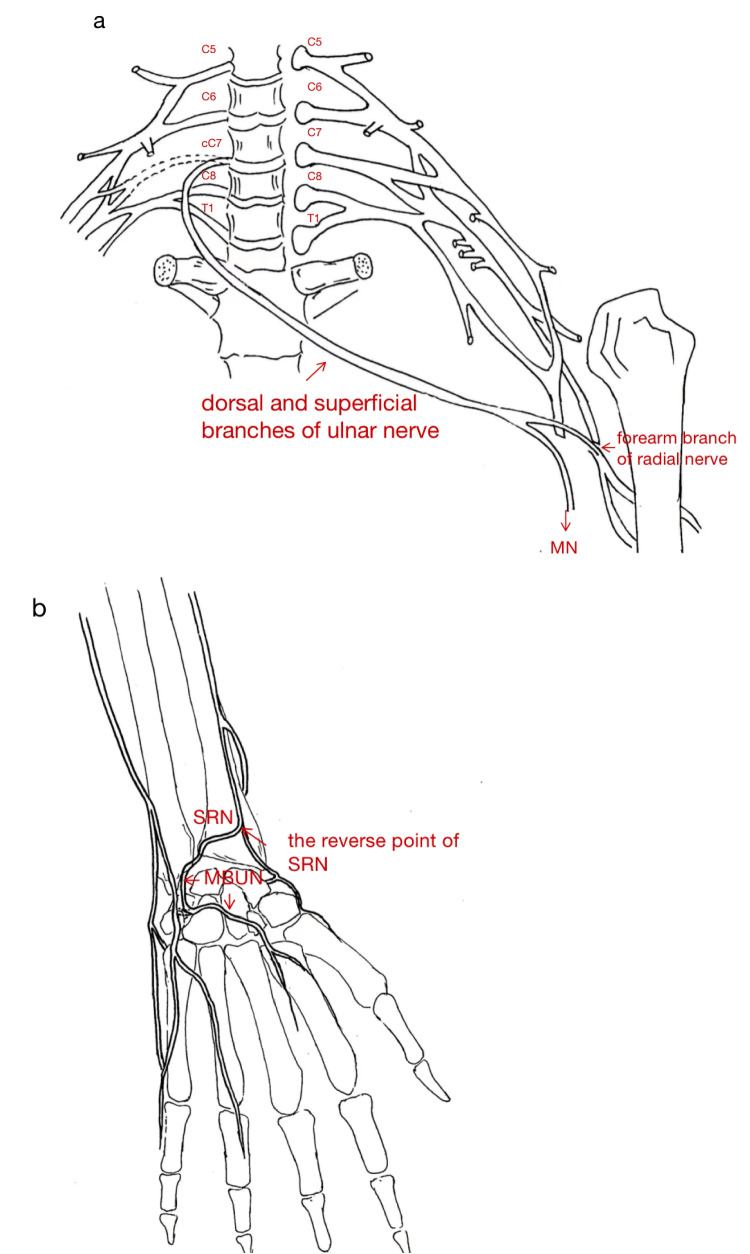
Modified Contralateral seventh cervical nerve root transfer surgery. **a** Dorsal and superficial branches of ulnar nerve on the injury side was used as graft. The Contralateral seventh cervical (cC7) was transferred to the median nerve (MN) and the forearm branch of radial nerve. **b** The superficial radial nerve (SRN) was transferred to the motor branch of ulnar nerve (MBUN)

Given the limitations of cC7, we developed a modified cC7 nerve transfer. In this procedure, the MBUN on the injured side was preserved, while the dorsal and superficial branches of the ulnar nerve were used as grafts to connect the recipient nerve and cC7, as in previous protocols [11, 18]. We also used the SRN to bridge the gap between the ulnar nerve (without MBUN) and the MBUN, enabling both the median and MBUN to be innervated (Fig. 1).

This study seeks to investigate the feasibility of this modified cC7 transfer in cadavers. The cC7 procedure was carried out as previously described [9, 14], given its extensive documentation. Thus, this study primarily focused on the anatomy of SRN and MBUN. We measured distances and diameters and counted the number of axons to assess the degree of compatibility between the SRN and MBUN. Although it is traditionally believed that the functions of intrinsic muscles have a slim chance of recovery [18], by preserving the MBUN, the possibility still exists.

## Materials and methods

This study utilized twenty upper extremities from 10 formalin-fixed cadavers (4 males and 6 females) supplied by the Department of Anatomy, Histology, and Embryology at Fudan University.

**Fig. 2:**
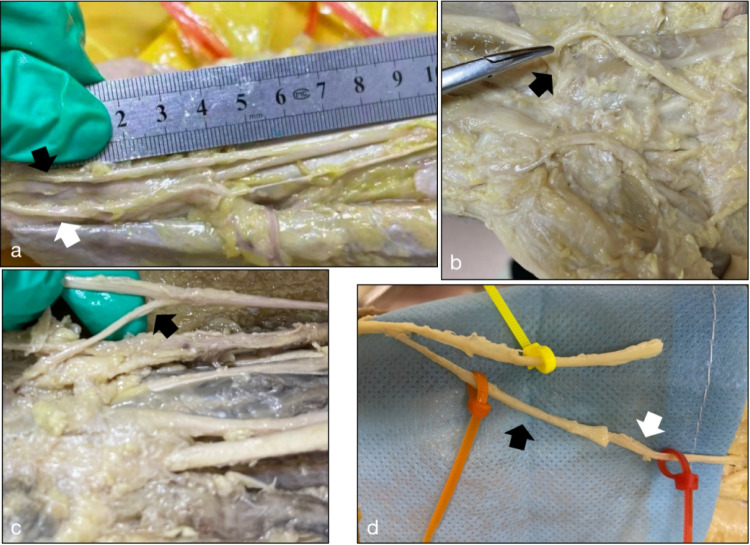
Anatomy of the median and ulnar nerves in the wrist. **a** The superficial radial nerve was ramified into a anterior branch (black arrow) and a posterior branch (white arrow). **b** The the motor branch of the ulnar nerve (black arrow) and superficial branch were identified. **c** The black arrow points to the dissected motor branch of ulnar nerve. **d** The superficial radial nerve (black arrow) and the motor branch of the ulnar nerve (white arrow) are prepared for tension free nerve repair

With the forearm in a supine position, dissection of the SRN and ulnar nerve was carried out. Starting from the distal third of the radial border of the forearm, a longitudinal incision was made, extending distally to the wrist flexion crease. The SRN was found to ramify into an anterior and a posterior branch. These finer branches were dissected more distally and transected just proximal to where smaller branches appeared (Fig. 2a). The ulnar nerve was exposed from the forearm towards the palm. Within Guyon's canal, the MBUN and the superficial branch were first identified (Fig. 2b). The MBUN was carefully dissociated intraneurally from distal to proximal until the fascicles mixed (Fig. 2c). The origin point of the sensory branch of the ulnar nerve was also identified.

Next, the distances from the reverse point of SRN to the wrist flexion crease, as well as to the coaptation point of SRN and MBUN, were measured with a vernier caliper. The maximum length of MBUN was measured, and the diameters of nerve branches and the number of axons were recorded. The feasibility of coaptation was evaluated by positioning the SRN close to the MBUN (Fig. 2d).

**Fig. 3:**
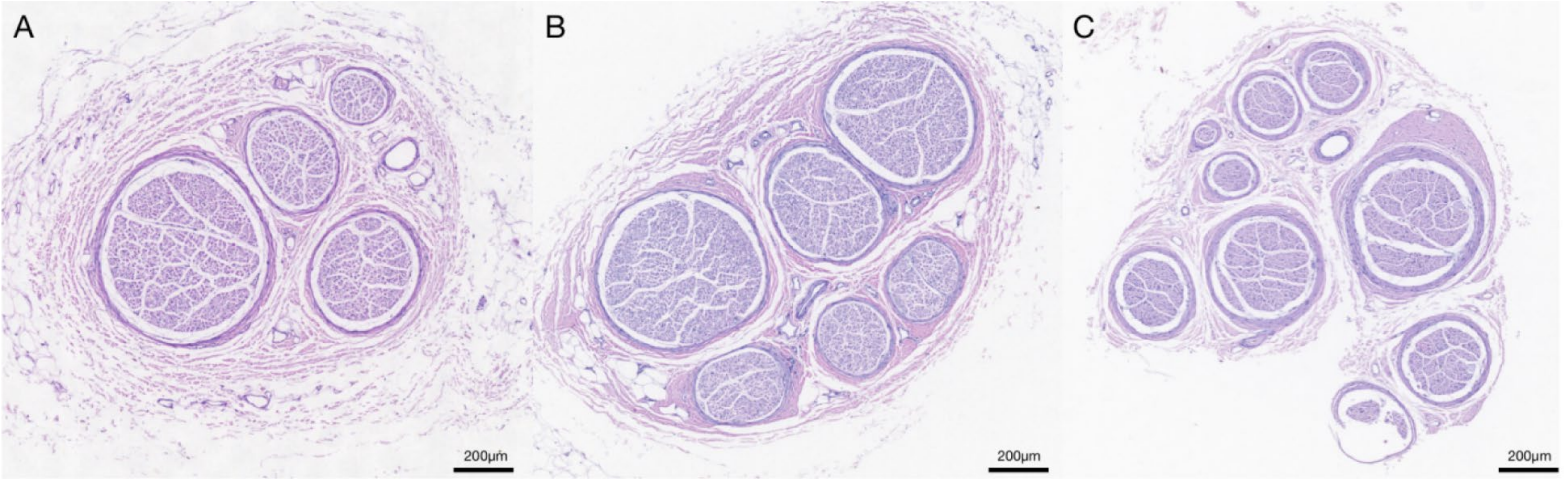
Cross section of anterior branch of SRN (**A**), posterior branch of SRN (**B**) and MBUN (**C**). SRN: superficial radial nerve, MBUN: the motor branch of the ulnar nerve

For axon count, nerve tissue was harvested, fixed with 10% formalin, dehydrated through a graded alcohol series, and embedded in paraffin blocks. Cross-sections were cut to a thickness of 0.5 μm and stained with hematoxylin and eosin. The total number of axons was examined under 200 × magnification with Leica Microsystems (LeicaDWLB2, Leica, Heidelberg, Germany) and analyzed using Image J by measuring the view area and axon counts (Fig. 3).

Finally, the nerve diameters and axon numbers of SRN and MBUN were analyzed at the site of coaptation.

### Statistic analysis

Data were represented as mean ± SD. All statistical analyses were carried out using SPSS 24.0 software (SPSS Inc., Chicago, IL, USA). Comparisons between groups were conducted using the t-test, with a two-tailed approach for statistical significance. A significance level (α) was set at 0.05, and *p*-values less than 0.05 were considered to denote statistical significance.

## Results

In all examined specimens, nerve coaptation was anatomically and technically feasible in a neutral wrist position without undue tension. The ulnar nerve divides into motor and sensory branches after crossing through Guyon’s tunnel. The maximum dissection length of the MBUN, from its point of original divergence to the point where fascicles intertwined, was 7.62 ± 1.03 cm, which did not exceed the dorsal sensory branch rise point. The distance from the reverse point of the SRN to the wrist flexion crease was 8.24 ± 1.80 cm, establishing the required exploration length of the SRN from distal to proximal. The distance from the reversal point of the SRN to the coaptation point was also 6.60 ± 1.75 cm, which was significantly shorter than the maximum length of the MBUN (Table 1).

**Table 1:** The distance from the reverse point of SRN to wrist flexion and to the coaptation point and maximum dissectable length of the MBUN

	**Distance (cm) ± SD**
reverse point of SRN to wrist flexion	8.24 ± 1.80^*^
reverse point of SRN to coaptation point	**6.60 ± 1.75**^**#**^
maximum dissectable length of the MBUN	**7.62 ± 1.03**

*SRN* superficial radial nerve, *MBUN* the motor branch of the ulnar nerve

^#^It was significantly shorter than the maximum length of the MBUN (*P* < 0.05)

^*^It determine the distance of superficial radial nerve needs to be explored from the distal to the proximal

The diameters of the anterior and posterior branches of the SRN and the MBUN were 1.56 ± 0.38 mm, 2.02 ± 0.41 mm, and 1.88 ± 0.42 mm, respectively (Table 2). The respective axon counts for the anterior and posterior branches of the SRN and the MBUN were 721.50 ± 138.2, 741.90 ± 171.34, and 1426.60 ± 331.39 (Table 3). The axon count for the MBUN was significantly greater than that of the anterior and posterior branches of the SRN (*p* < 0.05). The ratio of the axon count between the SRN and the MBUN was approximately 1:2.

**Table 2:** The mean diameter of the nerves

Nerve	Mean diameter (mm) ± SD
Anterior branch of SRN	**1.56 ± 0.38**
Posterior branch of SRN	**2.02 ± 0.41**
MBUN	**1.88 ± 0.42**

*SRN* superficial radial nerve, *MBUN* the motor branch of the ulnar nerve

**Table 3:** The number of fibers in each nerve

Nerve	Number of axons (mean ± SD)
Anterior branch of SRN	721.50 ± 138.22^*^
Posterior branch of SRN	741.90 ± 171.34^*^
MBUN	**1426.60 ± 331.39**

*SRN* superficial radial nerve, *MBUN* the motor branch of the ulnar nerve

^*^the axon number of the MBUN was also significantly greater than that of the Anterior and Posterior branch of SRN (*P* < 0.0001)

## Discussion

Contralateral c7 nerve transfer, introduced by Gu et al., has become a crucial treatment modality for total brachial plexus avulsion injuries, particularly when donor nerves are limited [10]. This technique has been globally adopted, yielding promising outcomes. Gao et al.'s report indicated that among 22 cases, recovery rates to M3 or greater were 68.18% (15 out of 22 patients) for wrist and finger flexors, 66.67% for biceps, and only 20% for triceps [7]. Terzis et al. reported total recovery rates of fair (M2 +  ~ M3), good (M3 +  ~ M4 −), and excellent (M4 +  ~ M5 −) in 56 cases, showing 74% for biceps, 57% for triceps, 62% for wrist and finger flexors, and 40% for wrist and finger extensors, respectively [20].

The function of intrinsic muscles, as the orchestrators of fine motor control, can significantly enhance the restoration of the affected limb. However, the conventional cC7 procedure involves harvesting the entire ulnar nerve segment as a graft to bridge the gap between the median nerve and cC7, which completely severs the physical connection between the ulnar-innervated intrinsic muscles and the MBUN. Consequently, the outcomes from the current surgical procedures remain sub-optimal.

The C7 nerve typically contains between 27,000–41,000 nerve fibers, providing ample axons to reconstruct more than one recipient nerve [11]. The cC7 nerve transfer could potentially be used to repair two recipient nerves without compromising the recovery of the median nerve [14]. However, in the traditional cC7 procedure, the median nerve is the only recipient nerve, which might lead to underutilization of the numerous fibers in C7.

Traditionally, it was thought that the ulnar nerve had little potential for repair due to factors such as the long-distance needed for nerve growth, slow speed of nerve regeneration, and atrophy of denervated intrinsic muscles. Furthermore, the atrophy of denervated intrinsic muscles occurs more rapidly than that of denervated arm muscles [23]. Therefore, the entire ulnar nerve was used as the graft.

However, recent research has challenged these views. For instance, Wang et al. reported reinnervation of the thenar muscle after repair of total brachial plexus avulsion injury with cC7 transfer to the median nerve in five cases, with four achieving strength of grade M2 and one achieving grade M1 in the abductor pollicis brevis [13]. Moreover, Yang et al. reported on 95 patients with total brachial plexus avulsion injury, all of whom underwent cC7 transfer surgery. Electromyography revealed motor unit potential (MUP) recovery of the abductor pollicis brevis in 53 patients [24].

The reports mentioned above highlight the limited potential for nerve regeneration due to long distances and slow speeds. However, it is noteworthy that the abductor pollicis brevis muscle is still capable of regenerating, which offers the possibility of reconstructing hand function in cases of total brachial plexus avulsion injury. Moreover, Yu et al. reported on 20 cases that underwent repair of the median nerve and MBUN using the bridge technique with the pedicled ulnar nerve and antebrachial cutaneous nerve. In two cases, a small amount of MUP was recorded in the dorsal interosseous muscle. Additionally, in one case, compound muscle action potential (CMAP) was observed in the abductor digiti minimi and dorsal interosseous muscle [25]. Their study not only illustrates the regenerative capability of the abductor pollicis brevis muscle but also demonstrates the potential for hand function restoration in brachial plexus avulsion injuries. Furthermore, Chen et al. performed a cC7 transfer procedure in rat model to preserve the deep branch of the ulnar nerve, which was then anastomosed with the anterior interosseous nerve to restore innervation of the ulnar nerve-controlled muscles [3]. The results of this study showed promising potential for enhancing intrinsic muscles function recovery. It is evident from these studies that there remains a possibility of recovery for the intrinsic muscles innervated by the MBUN. As a result, it is imperative to devise a method that preserves the MBUN in the affected limb. An anatomical study revealed that in cases of combined high median and ulnar nerve injuries, it is feasible to transfer the SRN to both the MBUN and the motor branch of the median nerve [15]. The SRN, being in close proximity to the ulnar nerve and having a constant path, provides an opportunity for direct sutures without the need for a graft. These features make the SRN an ideal donor nerve for the repair of the MBUN.

In the present study, all MBUN were fully separated from the main trunk of the ulnar nerve, and a sufficient length of MBUN was obtained for coaptation. This distance was significantly longer than the distance from the reverse point of the SRN to the coaptation point (*P* < 0.05). In all specimens, tension-free nerve coaptation could be performed with the wrist in a neutral position.

In our dissections, we found that the maximum length of the MBUN we could dissect—from its original divergence to the point where the fascicles mixed—was not longer than the point of emergence of the dorsal sensory branch. This finding suggests that the emergence point of the dorsal sensory branch could be used as a landmark indicating where ulnar nerve dissection should stop. Furthermore, after dissection, the proximal end of the SRN could be used as a pivot point for reversing the SRN towards the ulnar side for anastomosis with the MBUN, a process termed the "reverse point". The distance from the reverse point of the SRN to the wrist flexion crease was 8.20 ± 1.72 cm, suggesting this is the distance the SRN needs to be explored from distal to proximal.

The diameters of the anterior and posterior branches of the SRN and the MBUN were comparable, indicating no significant mismatch (Table 2). The ratio of axon numbers between the SRN and MBUN was about 1:2, aligning with the principle that the number of nerve fibers of the donor nerve should be at least 30% that of the recipient nerve [21].

This modified contralateral cC7 procedure has several potential advantages. Firstly, preservation of the MBUN could allow the reconstruction of intrinsic muscles innervated by both the median and ulnar nerves. Secondly, the largely preserved blood supply of the SRN could theoretically expedite nerve regeneration. Lastly, direct sutures may speed up the nerve regeneration process.

However, There are indeed several limitations to this study. The ulnar nerve, especially at the mixing point of the motor, superficial, and dorsal branches, is complex, making intraneural dissection time-consuming and demanding more surgical skill. Additionally, the regeneration of the MBUN would need to navigate through three nerve coaptation sites, potentially influencing recovery efficiency. Lastly, the number of nerve fibers in the ulnar nerve after MBUN removal is less than in the traditional procedure. The impact of this on functional recovery remains unclear and requires further evaluation. It is also unclear whether this modified procedure can effectively reconstruct intrinsic muscles—this will need to be verified through future clinical trials.

In clinical practice, a two-stage surgery is recommended when applying this modification. In the first stage, the MBUN is dissected from the main trunk of the ulnar nerve, and the superficial and dorsal branches are transected at the wrist level and drawn across the chest through a subcutaneous tunnel to the contralateral neck. The distal ends of the superficial and dorsal branches of the ulnar nerve are then anastomosed with the entire root contralateral cC7 nerve. At the wrist level, the MBUN is coapted to the SRN.

Zhao et al. reported the utilization of selective neurotization as a treatment method for brachial plexus palsy [26]. Similarly, Yang et al. employed micro-anatomical analysis to investigate the internal topography of fascicular groups within the main trunk of the radial nerve at the axillary level. Their findings indicated that the superficial radial nerve is situated on the lateral aspect of the main trunk of the radial nerve at the level of latissimus dorsi insertion in the axilla [12]. Based on these insights, in the second stage of the procedure, the ulnar nerve on the affected side was surgically severed at the axillary level and then connected with both the median nerve and the branch of the superficial radial nerve simultaneously to allow for functional restoration.

Nonetheless, rehabilitating the intrinsic muscles of patients with total brachial plexus avulsion injury remains a significant challenge in clinical practice. This study only validates the anatomical feasibility of the modification. Its effectiveness needs to be further supported by animal experiments and clinical trials.

In conclusion, transferring the SRN to the MBUN without needing a nerve graft is anatomically feasible. This study may provide a theoretical and practical foundation for modifying current rehabilitation strategies for total brachial plexus avulsion injuries.

## Data Availability

The datasets used and analysed during the current study are available from the corresponding author on reasonable request.

## Abbreviations

cC7: Contralateral seventh cervical
MBUN: Motor branch of the ulnar nerve
SRN: Superficial radial nerve
MUP: Motor unit potential
CMAP: Compound muscle action potential
